# High Dose Rate Brachytherapy as a Treatment Option in Endobronchial Tumors

**DOI:** 10.1155/2016/3086148

**Published:** 2016-07-14

**Authors:** Ali Hosni, Andrea Bezjak, Alexandra Rink, Kasia Czarnecka, Andrew McPartlin, Susan Patterson, Elantholiparameswaran Saibishkumar

**Affiliations:** ^1^Department of Radiation Oncology, Princess Margaret Cancer Centre, University of Toronto, 610 University Avenue, Toronto, ON, Canada M5G 2M9; ^2^Division of Respirology and Thoracic Surgery, University Health Network, Toronto, ON, Canada; ^3^Radiation Medicine Program, Princess Margaret Cancer Centre, Toronto, ON, Canada

## Abstract

*Purpose*. To report our experience with high dose rate endobronchial brachytherapy (HDR-EBBT) and to assess its efficacy and tolerability with possibility of its use in selected cases with curative intent.* Method*. Retrospective review of patients with endobronchial tumors treated at our institution in 2007–2013 with HDR-EBBT. Subjective response and treatment related toxicity were extracted from patients' records. Clinical response was evaluated by chest CT +/− bronchoscopy 2-3 months after treatment. Local control (LC) and overall survival (OS) were analyzed.* Results*. Overall 23 patients were identified. Ten patients were treated with curative intent, in 8 of them HDR-EBBT was combined with external beam radiotherapy. Short term palliation was as follows: dyspnea (13/15), cough (12/14), and hemoptysis (3/3). Seventeen patients were evaluated, of whom 9 (53%) showed complete response. Four patients developed local failure (only 1 of them treated with curative intent) and were salvaged with HDR-EBBT (*n* = 1), chemotherapy (*n* = 2), and laser (*n* = 1). Among patients treated with curative intent, the 2-year LC and OS were 89% and 67%, respectively, and 2 out of 4 deaths were cancer-related. Late toxicity included bronchial stenosis (*n* = 1). Only 1 patient had fatal hemoptysis and postmortem examination indicated local recurrence.* Conclusion*. HDR-EBBT is promising treatment with tolerable complication if used in properly selected patients.

## 1. Introduction

Endobronchial involvement can be seen in primary or recurrent lung cancer and lung metastases from other primary tumors. Curative and palliative approaches include external beam radiotherapy (EBRT), high dose rate endobronchial brachytherapy (HDR-EBBT), cryotherapy, laser therapy, photodynamic therapy, and endobronchial stent insertion [1].

HDR-EBBT successfully palliated symptoms in the majority of patients in several studies [2]. However, it also reported good outcomes in terms of local control (LC) and survival in definitive treatment of selected cases [3–6]. In this study, we report our experience with HDR-EBBT to assess its efficacy and tolerability in the treatment of patients with endobronchial tumors.

## 2. Materials and Methods

### 2.1. Patient Selection

After institutional research ethics board approval, a retrospective review of all patients with endobronchial (lung or metastatic) tumor (≥18 years old) with or without extrabronchial tumor, who had been treated in our institution between 2007 and 2013 with HDR-EBBT with either curative or palliative intent, alone or in combination with other treatment modalities, was conducted.

### 2.2. Treatment

HDR-EBBT was carried out on an outpatient basis. The depth of the prescribed dose was modified based on the diameter of the airways in curative cases: 10, 7, and 5 mm for tumors of the trachea, main bronchi, and lobar bronchi, respectively [7]. A margin of 1–1.5 cm on proximal and distal end of gross tumor volume was added to account for microscopic extension and planning uncertainties. A protective sheath (Fritz applicator) was used if possible to protect the bronchial mucosa from high doses and to immobilize the catheter containing the radioactive source.

In curative setting, HDR-EBBT was considered alone for in situ non-small cell lung cancer (NSCLC) or positive microscopic endobronchial margin, while invasive endobronchial NSCLC was treated with a combination of EBRT (40–45 Gy at 1.8–2 Gy/fraction) and HDR-EBBT (16 Gy in 2 sessions or 15 Gy in 3 sessions). For tumors with extension beyond the bronchus, with nodal disease or synchronous parenchymal tumor, EBRT delivered 60 Gy in 30 fractions for 6 weeks with HDR-EBBT of 10 Gy in 2 sessions, sequential or during the course of EBRT.

In palliative setting, patients were treated with HDR-EBBT of 10 Gy in 1 session or 14 Gy in 2 sessions with a week in between, either alone or in combination with EBRT of 20 Gy in 5 fractions or equivalent dose in a sequential approach.

### 2.3. Evaluation

Subjective response in terms of resolution of the pretreatment symptoms was extracted from patients' medical charts within 8 weeks from the first HDR-EBBT session and categorized as complete, partial, or no resolution. Clinical response was evaluated by bronchoscopy and/or chest CT at 2 to 3 months following the treatment. Follow-up evaluations were performed every 6 months and included chest CT and surveillance bronchoscopy; biopsies were taken in case of suspicious lesions.

### 2.4. Statistical Analysis

For curatively treated patients, the estimated rate of LC was calculated using the competing risk method, and overall survival (OS) was analyzed by Kaplan-Meier method.

## 3. Results

### 3.1. Patients' and Treatment Characteristics

Overall, 23 patients with endobronchial tumor were treated, 20 of them with primary or recurrent NSCLC and 3 patients with lung metastases (from colon cancer and thymoma). The patients' and tumor characteristics are summarized in Table 1. Treatment was individualised for each patient in relation to tumor stage, treatment intent, patient comorbidities, and previous treatment and in discussion at multidisciplinary cancer conference. As a part of integral approach to cancer treatment, HDR-EBBT was used in conjunction with other procedures, which are summarized in Table 2.

Ten patients were treated with curative intent, only 2 of them received brachytherapy (BT) alone (1 at the site of postoperative positive microscopic endobronchial margin and the other one had in situ NSCLC), while 8 patients received HDR-EBBT in combination with EBRT for endobronchial tumor without extrabronchial extension (*n* = 2) or with extrabronchial extension (*n* = 1), nodal disease (*n* = 4), or synchronous parenchymal tumor (*n* = 1).

Palliative HDR-EBBT was given for patients with endobronchial lung metastases (*n* = 3), stage IV NSCLC with endobronchial tumor (*n* = 5), for patients with tumor recurrence at previously irradiated area (*n* = 4, 3 of which had radical EBRT before and 1 had stereotactic lung radiotherapy), and in a patient with short life expectancy (*n* = 1).

### 3.2. Palliation Rate and Clinical Response

Twenty out of 23 patients had documentation in the chart regarding subjective symptom response. Initial symptoms were dyspnea (65%), cough (61%), hemoptysis (13%), and chest pain and wheeze (each, 9%). Most patients had either complete or partial palliation within 8 weeks from initial BT session (Table 3).

Clinical response was assessed in 17 patients by CT, with (*n* = 7) or without (*n* = 10) bronchoscopy, and showed 9 out of 17 patients (53%) with complete response (CR) and 8/17 (47%) with partial response (PR). Six patients were not evaluated by CT or bronchoscopy, five because the treatment intent was symptom palliation and one who died 2 months after HDR-EBBT.

### 3.3. Local Control and Overall Survival

Among patients treated with curative intent, the median follow-up was 17 months and the 2-year LC was 89% (95% CI: 79–99%); only 1 patient had local failure (LF) at 7 months and was treated with chemotherapy and subsequently developed multiple-site distant metastases, while, in patients treated with palliative intent, 3 had LF at 7, 12, and 36 months and were further palliated with another palliative HDR-EBBT of 5 Gy in 1 session (*n* = 1), chemotherapy (*n* = 1), and laser therapy (*n* = 1). For the 4 patients in the entire group who developed LF, none of them had complete response after HDR-EBBT.

**Figure 1:**
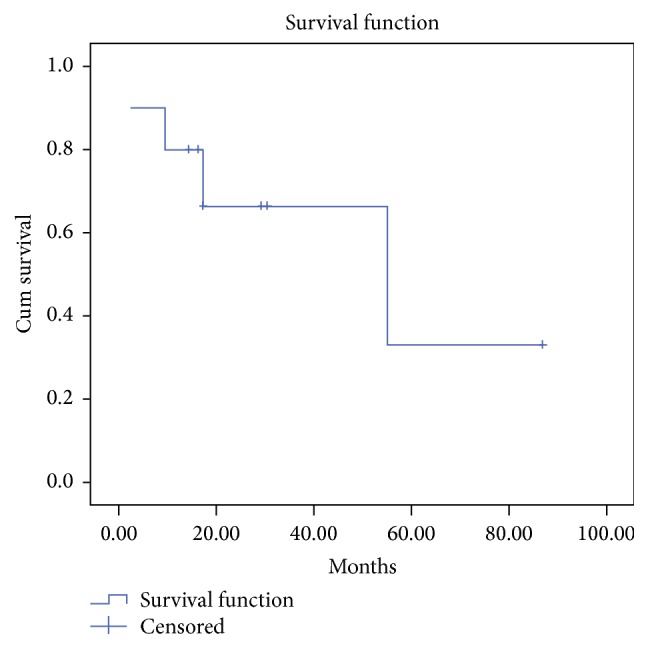
Overall survival in patients treated with high dose rate endobronchial brachytherapy with curative intent.

The 2-year OS for curatively treated patients was 67% (95% CI: 51–83%) (Figure 1); four patients died: 2 who were endobronchial cancer-related and 2 with unknown cause of death, of whom 1 patient died within 2 months of HDR-EBBT but death was deemed not treatment related.

### 3.4. Complications

No serious complications were recorded during HDR-EBBT procedure. Eighteen patients were alive 6 months after therapy, of whom only 1 patient had bronchial stenosis. Fatal hemoptysis was reported in one patient (that patient was previously treated with lung stereotactic radiotherapy with 54 Gy in 3 fractions and postmortem examination indicated recurrent disease in the lung).

## 4. Discussion

Therapeutic modalities for endobronchial tumors include EBRT, HDR-EBBT, laser therapy, phototherapy, endobronchial stent insertion, and combinations of these techniques [1]. Low dose rate endobronchial brachytherapy (LDR-EBBT) has been used, but a catheter must be left in place for 1–3 days, with the attendant risk of displacement thus requiring hospitalisation of the patient during treatment and surveillance. For this reason, LDR-EBBT has fallen into disuse. On the other hand, HDR-EBBT neither necessitates keeping the catheter in place for long time nor requires hospitalisation [8]. HDR-EBBT has been used with different fractionation regimens either with palliative or curative intent of such tumors [3–10].

A 2012 Cochrane review of palliative HDR-EBBT for NSCLC analyzed 14 randomized controlled trials (RCTs) involving 953 participants but a meta-analysis was not done due to heterogeneity in the patient characteristics, radiotherapy doses used, and outcomes measured [2]. The authors concluded that EBRT is more effective for palliation of symptoms than HDR-EBBT. Their findings did not provide conclusive evidence to recommend addition of HDR-EBBT to EBRT in palliative setting. A currently accruing randomized study, OCOG-2011-BRACHY multicentre RCT (NCT01351116), may provide an answer to this question, by randomizing patients to EBRT +/− HDR-EBBT, with the primary outcome being patient-rated symptoms [11].

A variety of fractionation schemes have been used in palliative HDR-EBBT, which precludes meaningful comparison between studies [2]. Skowronek et al. [9] randomized 648 patients to receive 3 fractions of 7.5 Gy (time interval: 1 week, *n* = 303) or single fraction of 10 Gy (*n* = 345). The two treatment protocols showed similar palliative efficiency. There had always been a concern about association between fraction dose and fatal hemoptysis. The largest study was a retrospective review of 938 patients treated with EBRT and/or HDR-EBBT, and 101 patients (10.8%) died from massive hemoptysis. In the multivariable analysis, a single dose of 15 Gy HDR-EBBT was the most important prognostic factor for massive hemoptysis [10]. In our institution we use 10 Gy in 1 session or 14 Gy in 2 sessions with a week in between in palliative setting, and only 1 patient developed massive hemoptysis; however the postmortem examination indicated local tumor recurrence.

The value of HDR-EBBT as a curative treatment is not widely accepted, in either definitive or postoperative setting. The use of HDR-EBBT as a sole treatment of endobronchial NSCLC without nodal or visceral metastases was reported by Hennequin et al. [3]. Treatment consisted of six fractions of 5 or 7 Gy. The complete histologic response rate was 59%. At 5 years, LC and OS were 52% and 24%, respectively. Factors associated with LF were large tumor volume and previous endoscopic treatment. Five deaths were attributed to the HDR-EBBT procedure (two from fatal hemoptysis and three from bronchial necrosis).

Muto et al. [4] evaluated three different HDR-EBBT regimens (10 Gy in one fraction, 14 Gy in two fractions, or 21 Gy in three fractions) given concomitantly with EBRT (60 Gy in 30 fractions) in a prospective study with 320 patients with stage IIIA-IIIB NSCLC with endobronchial involvement. Mean OS was 11.1 months and was similar for the three groups. For the patients treated with three fractions of HDR-EBBT plus EBRT, a smaller number of side effects occurred, while relief from symptoms was similar for the three groups.

In most of studies, the dose of HDR-EBBT is prescribed at fixed depth regardless of the site of the tumor. As the intention is to prescribe the dose at bronchial mucosa, it is important to modify the prescription depth based on the diameter of the airways in order to avoid excessive dose to mucosa and underdosage to endobronchial tumor. Kawamura et al. [7] treated 16 endobronchial lesions with HDR-EBBT in curative intent with 5 Gy/fraction. Ten lesions were treated with HDR-EBBT of 20 Gy combined with EBRT of 45 Gy, and 6 lesions were treated with HDR-EBBT alone of 25 Gy. The depth of prescription was provisionally standardized of 10 mm for the trachea, 7 mm for the main bronchi, and 5 mm for the lobar and segmental bronchi. The 2-year LC and OS were 86% and 92%, respectively. Complications greater than grade 2 were not observed except for one grade 3 dyspnea.

Our experience, albeit with relatively small number of patients, is consistent with other literature that HDR-EBBT can provide effective palliation and can even be used in curative setting in properly selected patients with endobronchial tumors. In our curative protocol, the 2-year LC and OS were 89% and 67%, respectively. We believe that the therapeutic ratio of HDR-EBBT could be maximized by proper fractionation schedule and modifying the depth of the prescribed dose according to the diameter of the airway. Another important factor is to use the protective applicator whenever airways allow protecting the bronchial mucosa from high doses and immobilizing the catheter containing the radioactive source. Avoiding very high dose per fraction and combining EBRT also help in maintaining dose homogeneity; however further research is required to determine incremental benefit of addition of HDR-EBBT to EBRT especially in curative setting.

## 5. Conclusion

HDR-EBBT is a promising palliative and curative treatment with tolerable complication if used in properly selected patients with proper fractionation schedule; however its combination with other treatment modalities needs further studies.

## Figures and Tables

**Table 1:** Patients' and tumor characteristics.

Characteristic	Value
Median age (range), year	70 (37–92)

Median follow-up (range), months	17 (1.5–86)

Gender	
Male	14 (61%)
Female	9 (39%)

Smoking history	
Yes	13 (65%)
No	7 (35%)
Unknown	3

ECOG performance status	
0	1 (4%)
1	17 (74%)
2	5 (22%)

Histology	
Primary lung tumor	20 (87%)
SCC	9 (39%)
Adenocarcinoma	9 (39%)
Others	2 (9%)
Lung metastases	3 (13%)
Colon adenocarcinoma	2 (9%)
Thymoma	1 (4%)

Site	
Central (trachea, main bronchi)	11 (48%)
Peripheral (lobar bronchi)	12 (52%)

Previous lung tumor	
Yes	16 (70%)
No	7 (30%)

Treatment to previous lung tumor	
Surgery	7 (30%)
Lobectomy	4 (17%)
Pneumonectomy	1 (4%)
Metastasectomy	2 (9%)
Radiotherapy	10 (43%)
Chemotherapy	9 (39%)
Photodynamic therapy	1 (4%)

Tumor visible on CT	18 (78%)

EBRT: external beam radiotherapy.

**Table 2:** Treatments administered to the treated endobronchial tumors.

Treatment	Curative *n* = 10	Palliative *n* = 13
HDR-EBBT alone	2	6
HDR-EBBT + EBRT	5	1
HDR-EBBT + chemotherapy	0	2
HDR-EBBT + EBRT + chemotherapy	2	2
HDR-EBBT + surgery + EBRT	1	2

HDR-EBBT: high dose rate endobronchial brachytherapy; EBRT: external beam radiotherapy.

**Table 3:** Short term palliation rate.

	Before BT *n* (%)	CR *n* (%)	PR *n* (%)
Dyspnea	15 (65)	7 (47)	6 (40)
Cough	14 (61)	8 (57)	4 (29)
Hemoptysis	3 (13)	2 (67)	1 (33)
Chest pain	2 (9)	1 (50)	1 (50)
Wheeze	2 (9)	1 (50)	1 (50)

BT: brachytherapy, CR: complete resolution, and PR: partial resolution.

## References

[1] Celebioglu B., Gurkan O. U., Erdogan S. (2002). High dose rate endobronchial brachytherapy effectively palliates symptoms due to inoperable lung cancer. *Japanese Journal of Clinical Oncology*.

[2] Reveiz L., Rueda J.-R., Cardona A. F. (2012). Palliative endobronchial brachytherapy for non-small cell lung cancer. *Cochrane Database of Systematic Reviews*.

[3] Hennequin C., Bleichner O., Trédaniel J. (2007). Long-term results of endobronchial brachytherapy: a curative treatment?. *International Journal of Radiation Oncology, Biology, Physics*.

[4] Muto P., Ravo V., Panelli G., Liguori G., Fraioli G. (2000). High-dose rate brachytherapy of bronchial cancer: treatment optimization using three schemes of therapy. *Oncologist*.

[5] Skowronek J., Piorunek T., Kanikowski M., Chicheł A., Bieleda G. (2013). Definitive high-dose-rate endobronchial brachytherapy of bronchial stump for lung cancer after surgery. *Brachytherapy*.

[6] Anacak Y., Mogulkoc N., Ozkok S., Goksel T., Haydaroglu A., Bayindir U. (2001). High dose rate endobronchial brachytherapy in combination with external beam radiotherapy for stage III non-small cell lung cancer. *Lung Cancer*.

[7] Kawamura H., Ebara T., Katoh H. (2012). Long-term results of curative intraluminal high dose rate brachytherapy for endobronchial carcinoma. *Radiation Oncology*.

[8] Villanueva A. G., Lo T. C. M., Beamis J. F. (1995). Endobronchial brachytherapy. *Clinics in Chest Medicine*.

[9] Skowronek J., Kubaszewska M., Kanikowski M., Chicheł A., Młynarczyk W. (2009). HDR endobronchial brachytherapy (HDRBT) in the management of advanced lung cancer—comparison of two different dose schedules. *Radiotherapy & Oncology*.

[10] Langendijk J. A., Tjwa M. K. T., de Jong J. M. A., Ten Velde G. P. M., Wouters E. F. M. (1998). Massive haemoptysis after radiotherapy in inoperable non-small cell lung carcinoma: is endobronchial brachytherapy really a risk factor?. *Radiotherapy and Oncology*.

[11] https://clinicaltrials.gov/ct2/show/NCT01351116.

